# Supplementary material for: The causal role of circulating inflammatory markers in osteoporosis: a bidirectional Mendelian randomized study
Source: Front Immunol. 2024 Jul 18;15:1412298. doi: 10.3389/fimmu.2024.1412298 (PMC11291241; doi:10.3389/fimmu.2024.1412298)
Supplement: Supplementary file 1 [file DataSheet_1.zip › supplementary material/OSTEOPOROSIS-CIM/GCST90274835.tsv.gz_leave_one_out.pdf]

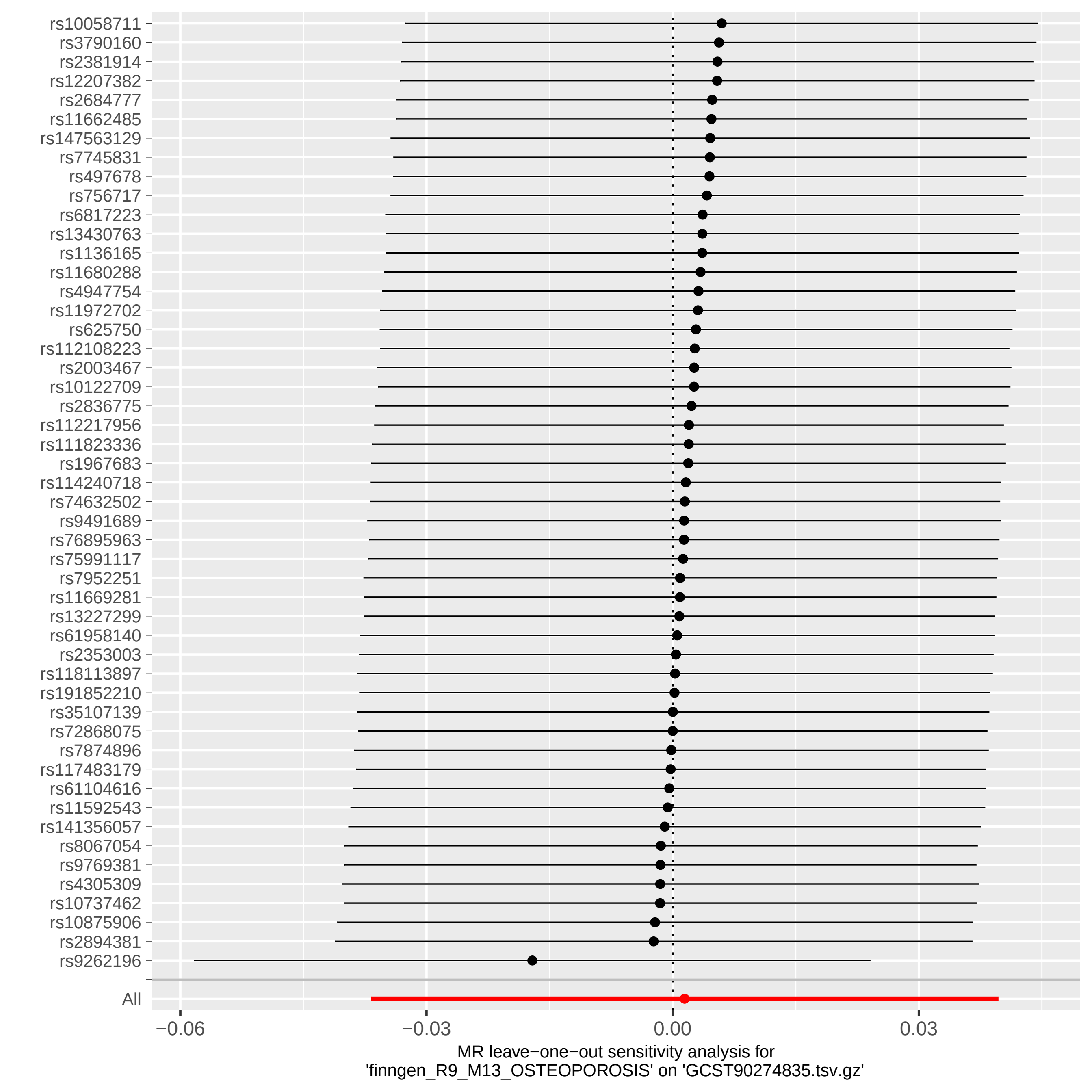
rs10058711
rs3790160
rs2381914
rs12207382
rs2684777
rs11662485
rs147563129
rs7745831
rs497678
rs756717
rs6817223
rs13430763
rs1136165
rs11680288
rs4947754
rs11972702
rs625750
rs112108223
rs2003467
rs10122709
rs2836775
rs112217956
rs111823336
rs1967683
rs114240718
rs74632502
rs9491689
rs76895963
rs75991117
rs7952251
rs11669281
rs13227299
rs61958140
rs2353003
rs118113897
rs191852210
rs35107139
rs72868075
rs7874896
rs117483179
rs61104616
rs11592543
rs141356057
rs8067054
rs9769381
rs4305309
rs10737462
rs10875906
rs2894381
rs9262196
All
−0.06
−0.03
0.00
0.03
MR leave−one−out sensitivity analysis for
'finngen_R9_M13_OSTEOPOROSIS' on 'GCST90274835.tsv.gz'
